# Clinical usefulness of polygenic risk scores in risk prediction models for lung cancer screening and lung nodule management

**DOI:** 10.1016/j.tranon.2026.102771

**Published:** 2026-04-15

**Authors:** Véronique Boumtje, Zhonglin Li, Nathalie Gaudreault, Victoria Saavedra Armero, Dominique K. Boudreau, Sophie Plante, Sabrina Biardel, Aida Eslami, Simon Martel, Sébastien Thériault, Philippe Joubert, Yohan Bossé

**Affiliations:** a Institut universitaire de cardiologie et de pneumologie de Québec – Université Laval, Quebec City, Canada; b Department of Social and Preventive Medicine, Université Laval, Quebec City, Canada; c Department of Molecular Medicine, Université Laval, Quebec City, Canada

**Keywords:** Polygenic susceptibility to lung cancer, Lung cancer screening, Lung nodule management

## Abstract

**Background:**

Lung cancer mortality decreases as a result of low-dose computed tomography (LDCT) screening, but suffers from low uptake and high false-positive rates. The impact of integrating genetic risk using a polygenic risk score (PRS) to optimize lung cancer screening remains underexplored.

**Methods:**

We developed a genome-wide PRS and evaluated its performance in pre- and post-screening contexts. Screening eligibility was assessed using two UK Biobank (UKB) subsets: UKB_PLCO_ (n = 8957; PLCOm2012norace risk ≥2%) and UKB_ScreeningCriteria_ (n = 74,024 meeting screening eligibility criteria). To evaluate nodule management, we used a cohort of 669 ever-smokers with PLCO ≥2% and Lung-RADS score ≥3, referred to as SYNERGIQC_PLCO_LungRADS_. Multivariable Cox models, time-dependent area under the curve (AUC), and decision curve analyses (DCA) evaluated association, discrimination, and clinical net benefit.

**Results:**

In UKB_PLCO_, the PRS was associated with a hazard ratio (HR) of 1.18 per standard deviation. In UKB_ScreeningCriteria_, PRS showed HR of 1.34. Adding PRS to the PLCOm2012 model improved discrimination (AUC: 0.707 vs 0.696; P = 6.85e^−27^[likelihood ratio test]) and correctly reclassified 9.2% of incident lung cancer cases. Six-year absolute risks stratified by PRS deciles indicate 3.1-fold increase in the top compared to the bottom decile. In SYNERGIQC_PLCO_LungRADS_, HR_PRS_ was 1.22. In this context, DCA indicated a modest net benefit for decision thresholds between 10% and 30%.

**Conclusion:**

PRS in lung cancer reveals context-dependent net benefit across studied populations. Although PRS adds limited value for determining screening eligibility, it may help reclassify borderline individuals and inform decisions regarding closer follow-up or invasive diagnostic procedures for high-risk screened groups.

## Highlights

• Polygenic risk score utility in real-world lung cancer screening remains underexplored.
• Polygenic risk score predicted incident lung cancer in both pre-screening populations and high-risk post-screening cohorts.
• Polygenic risk score offers context-dependent clinical net benefit.

## Introduction

Lung cancer remains the leading cause of cancer-related mortality worldwide, with most cases diagnosed at an advanced stage when curative treatment options are limited [1]. Early detection through low-dose computed tomography (LDCT) screening reduces mortality by 20–24% in high-risk populations, as demonstrated by the national lung screening trial (NLST) and NELSON trial [2,3]. However, real-world implementation has been hindered by low uptake, high false-positive rates leading to unnecessary investigations and treatment of benign nodules, and overdiagnosis, which results in the detection of small, non-progressive cancers that would not cause clinical symptoms or death. The limitations of current risk targeting approaches are further highlighted by ever-smokers who fall just above or below clinical screening thresholds [4], as well as individuals who have never smoked but are at high risk of developing lung cancer due to inherited susceptibility [5].

Risk prediction models such as the prostate, lung, colorectal, and ovarian cancer screening trial model 2012 (PLCOm2012) [6], improve screening eligibility [7]. Similarly, the lung computed tomography screening reporting and data system (Lung-RADS) [8] provides the standard protocol for managing lung nodules detected during screening. Still, PLCOm2012 and Lung-RADS do not account for measured genetic factors. In the case of PLCOm2012, the model relies on family history as an indirect proxy for inherited susceptibility, which may explain inter-individual variability in lung cancer risk [9].

A personalized, integrative approach using genetic information and existing risk prediction models might address these limitations [10]. Polygenic risk scores (PRS), derived from genome-wide association studies (GWAS), offer a quantitative approach to estimate inherited risk by aggregating common germline variants [11]. For lung cancer, several susceptibility loci have been identified across diverse populations [12], and recent studies suggest that PRS adds independent predictive value beyond traditional clinical factors [11,[13], [14], [15], [16]]. Yet, their utility in real-world screening contexts, especially when combined with validated risk prediction models, remains underexplored. In lung cancer screening programs, it remains unclear how individuals with low or high genetic risk, defined by the PRS could be integrated into the PLCOm2012 and Lung-RADS systems to optimize both the screening eligibility and the clinical workup of indeterminate nodules. For instance, individuals with high genetic risk may benefit from LDCT even with low or no smoking exposure, whereas those with low genetic risk may require greater smoking exposure to achieve a similar screening benefit. Similarly, after screening and given the same Lung-RADS score, individuals with a high PRS might warrant earlier follow-up or biopsy to detect fast-growing tumors, while those with a low PRS could potentially be reclassified for annual screening.

In this study, we evaluate the statistical added value of integrating a genome-wide PRS into risk models for lung cancer screening and pulmonary nodules management. We leverage two prospective cohorts: UK Biobank (UKB) participants meeting screening eligibility criteria, and SYNERGIQC [17] cohort, derived from a lung cancer screening initiative in the province of Quebec. The first objective is to evaluate the PRS's ability to predict the risk of lung cancer in the pre-screening cohort. We assess whether integrating the PRS improves models’ risk discrimination and time-to-diagnosis prediction, with a specific focus on clinical decision thresholds, such as six-year lung cancer risk exceeding 2%. We further determine the PRS’s capacity to correctly reclassify individuals across the eligibility cut-off point by calculating the category-based net reclassification index (NRI) and the clinical net benefit. We hypothesize that the PRS will enhance screening eligibility. The second objective is to assess the PRS's utility for risk stratification in the post-screening population. We hypothesize that the PRS will improve risk stratification for individuals with indeterminate pulmonary nodules.

## Methods

### Study design

We leveraged a prospective study including incident cases of lung cancer (UKB) and the provincial lung cancer screening program (SYNERGIQC). We constructed a pan-genomic PRS for lung cancer in UKB and SYNERGIQC. We evaluated the PRS clinical validity at two critical time points by modelling three decision-making contexts: UKB_ScreeningCriteria_ and UKB_PLCO_ as the pre-screening context and SYNERGIQC_PLCO_LungRADS_ as the post-screening context.

### Study populations

#### UK Biobank

We used UKB, a prospective cohort with a range of health-related outcomes, as the screening context population [18]. From 502,166 available participants aged 37–73 years, we excluded those who were not of European ancestry (n = 92,783), those without genotype data (n = 485), and those with cancer at baseline (n = 191). Only ever-smokers aged 55–73 with ≥20 years of smoking history and ≤15 years since cessation were included. These criteria ensured that UKB participants were comparable in eligibility and genetic ancestry to participants in the lung cancer screening program in the province of Quebec. As a result, 74,024 participants met the screening eligibility criteria, henceforth referred as “UKB_ScreeningCriteria_”. A high clinical risk subgroup (n = 8957) was further defined by a PLCOm2012norace risk score ≥2%, to match the Quebec lung cancer screening program. This subgroup is henceforth referred as “UKB_PLCO_”. To calculate the PLCOm2012norace in UKB, we applied the equations used in Pasquinelli et al. [19] (**Appendix A**). In UKB, missing data were imputed using k-nearest neighbors (kNN; k = 10) algorithm [20]. Variables related to smoking had the highest proportion of missing value, but this was below 3% of the population. We converted educational levels to its equivalent in the cohort, while other variables, including family history, smoking status, and the presence of chronic obstructive pulmonary disease (COPD), remained unchanged.

## *SYNERGIQC*

SYNERGIQC originates from the Quebec lung cancer screening program [17], a provincial multicenter pilot-study launched in 2021 for ever-smokers aged 55–74. The program initially enrolled participants, of whom 3542 were classified as high-risk based on a PLCOm2012norace risk score of ≥2%. This risk threshold was chosen according to the PanCan model [21], the precursor to the PLCOm2012 model validated in Canada [22]. From this high-risk group, participants were selected for the SYNERGIQC project, 2974 provided blood samples for research purposes to the AIRS biobank at the IUCPQ and CUSM sites. In SYNERGIQC, blood samples were collected during the first or second visit following enrolment in the screening program. As part of the Quebec lung cancer screening program, the Lung-RADS grading system (version 2022) was used to guide management decisions, such as close follow-up with LDCT or biopsy, consistent with previous reports [23]. In the current study, the subset of interest, referred to as “SYNERGIQC_PLCO_LungRADS_”, was selected for genetic analysis. This subset modelled the context of indeterminate pulmonary nodules and included participants with at least one abnormal LDCT scan at screening. These participants were classified as Lung-RADS 3+ and were therefore considered to have an indeterminate pulmonary nodule (n = 669). SYNERGIQC_PLCO_LungRADS_ data were collected locally, so all variables had no missing data.

### Outcome definition

Incident lung cancer was defined by pathological confirmation or LDCT diagnosis in SYNERGIQC. For UKB, ICD9 and ICD10 related codes were used as described previously [24]. **Supplementary Table 1** shows UKB data fields and codes for defining lung cancer cases. At the time of data analysis, the median follow-up was 13.7 years for UKB and 2.8 years for SYNERGIQC. **Supplementary Table 2** presents the comparative overview of the SYNERGIQC and the UKB cohorts.

### Genotyping and imputation

#### SYNERGIQC

Genotyping was performed using the Illumina global screening array (GSA) version 3 BeadChip with the multi-disease (MD) drop-in panel, which adds approximately 50,000 disease-specific genetic markers. Quality control (QC) involved the exclusion of low-quality genetic variants with 10th percentile of Illumina GenCall score ≤ 0.1, a call rate < 0.97%, and a minor allele frequency (MAF) of <1%. Samples with missing genotypes were excluded, and Hardy-Weinberg equilibrium P < 1e-7 was used to evaluate genotype errors. Samples with sex discordance, duplication or non-European ancestry (via principal component analysis (PCA)) were also excluded. After QC, 669 samples and ∼511,000 variants on autosomal chromosomes remained for imputation. **Supplementary Table 3** provides a summary of genotyping QCs for variants and samples. Ungenotyped single nucleotide polymorphisms (SNPs) were imputed via the TOPMed imputation server (minimac4, GRCh38). Variants with INFO <0.3 or MAF <0.001 were excluded. SNPs were harmonized to HapMap Phase 3 variants and lifted over to GRCh37 for matching with the reference panel used for variant selection. A total of 1,045,282 variants were selected for PRS calculation.

#### UK Biobank

Genotyping was performed on the Affymetrix UK BiLEVE Axiom and Axiom arrays. Imputation used the haplotype reference consortium (HRC) and UK10K+1000 Genomes reference panels. We excluded individuals with sex discordance, non-European ancestry, or samples with excess third-degree relatives (>10). Variants with INFO <0.3 or MAF <0.0001 were excluded. A total of 408,422 participants of European ancestry were included in the analysis.

#### Polygenic risk score construction

We constructed genome-wide PRS using summary statistics from a previously reported lung cancer GWAS including 29,266 cases and 56,450 controls of European ancestry [25]. The LDpred2-auto algorithm (R package bigsnpr v1.12.18), which does not require any validation set, was applied using linkage disequilibrium (LD) estimated from 408,422 UKB individuals. This method allows the target sample to also act as a reference panel for the LD matrix, helping to avoid overfitting, as long as the sample size is sufficient and representative of the GWAS study population. The PRS was calculated as a weighted sum of risk alleles, and standardized within each cohort. **Supplementary Figure 1** illustrates the workflow.

### Risk modeling and statistical analyses

#### Predictive models

We developed multivariable Cox proportional hazards models (R package survival v3.8–3) to assess the predictive value of PRS, PLCOm2012norace, and their combination. Models were adjusted for age, sex, and the top ten genetic principal components (PCs). The study end date was October 25, 2022 for UKB and May 27, 2025 for SYNERGIQC.

#### Discrimination

Discriminatory performance was assessed using the area under the receiver operating characteristic curve (AUC) and Harrell’s concordance index (C-index), which measure a model's ability to distinguish between individuals who experienced the event earlier and those who experienced it later. Time-dependent AUCs were calculated at clinically relevant horizons (2 and 5 years) using inverse probability of censoring weighting [26], implemented via the R package timeROC v0.4.

#### Risk stratification and cut-offs

We applied the Youden index to derive optimal PRS thresholds for dichotomizing participants into high and low genetic risk groups [27]. These were compared to commonly used percentile-based thresholds (PRS80, PRS90). Kaplan-Meier diagnosis-free probability and log-rank tests evaluated time-to-diagnosis differences.

#### Reclassification indices and net benefit

To evaluate the clinical validity of the PRS models for lung cancer, we calculated the six-year absolute risk of lung cancer in UKB by combining the Cox proportional hazards model coefficients with the Efron [28] estimator for the baseline hazard function (R package riskRegression v12.21). We then compared the models with and without the PRS using the category-based net reclassification index only in UKB_ScreeningCriteria_.

We quantified the clinical utility of PRS risk models applied to each study population with a decision curve analysis (R package rmda v1.6) across a range of lung cancer risk threshold probabilities (0–30%). In UKB, DCA was calculated from six-year absolute risk models, and the net benefits were compared between models with or without the PRS, either as a continuous variable or dichotomized using the Youden threshold. In SYNERGIQC_PLCO_LungRADS_, DCA was based on the Cox proportional hazards model, including the PRS Youden, to assess the net benefit of incorporating PRS for lung nodule malignancy status. Net benefit was calculated as the true-positive rate minus the false-positive rate based on the lung cancer risk threshold probabilities. It considers both the benefits of correctly identifying incident lung cancer (true positives) and the harms of intervening with individuals who do not yet have lung cancer (false positives).

#### Classification accuracy and threshold optimization

The performance of the lung cancer risk prediction models was evaluated by comparing their predicted values to the actual values for each cohort. The models were trained using 70% of the population and optimism-corrected discrimination estimates were derived using internal validation with 200 bootstrap resamples of the training set. Finally, the performance of the models on the remaining 30% of the population was evaluated. Confusion matrix was performed using the R package caret v7.0–1.

### Ethical approvals

UK Biobank received approval from the British National Health Service, North West - Haydock Research Ethics Committee (16/NW/0274). The SYNERGIQC research project, was approved by the IUCPQ ethics committee (2022–3752). All participants of UKB and SYNERGIQC provided informed consent at the baseline assessment.

### Data availability

The present analyses were performed as part of UK Biobank data application 25,205. The genome-wide PRS developed in this study is available in the PGS Catalog (www.pgscatalog.org, score ID: PGS005397). ILCCO summary statistics [25] are available in the GWAS catalog under access number GCST004748.

## Results

### Study cohorts and baseline characteristics

We analyzed 74,024 ever-smokers from the UK Biobank, representing a pre-screening population, of whom 8957 met the eligibility criteria for lung cancer screening according to the PLCOm2012norace model (≥ 2%) as well as 669 clinically high-risk post-screening participants from the SYNERGIQC cohort. Baseline characteristics are summarized in **Supplementary Table 4**. In the UKB_ScreeningCriteria_ cohort (n = 74,024), 3193 incident lung cancers (4.3%) were documented during a median follow-up of 13.7 years. In the UKB_PLCO_ clinical selected subset (n = 8957), 1124 incident lung cancers (12.5%) were recorded. In both UKB subsets, lung cancer cases had higher smoking exposure and were more frequently afflicted by COPD (**Supplementary Table 4**). A higher proportion of males was also observed among lung cancer cases, but this did not reach statistical significance in UKB_PLCO_. Age differed between the two groups in UKB subsets, but in opposite directions in UKB_PLCO_ and UKB_ScreeningCriteria_, which we considered not clinically meaningful given the small mean differences between the groups.

SYNERGIQC_PLCO_LungRADS_ consisted of 669 high-risk post-screening ever-smokers, with a mean age of 65.1 ± 4.8 years and 47.8% male. At the time of data analysis, the median follow-up was 2.8 years (interquartile range 1.9–3.4), and 123 participants (18.4%) had a diagnosis of lung cancer. Of these 116 patients (94.3%) had pathological confirmation, while the remaining seven (5.7%) were diagnosed based solely on imaging, as obtaining a tissue sample for pathological analysis was not possible. SYNERGIQC_PLCO_LungRADS_ participants diagnosed with lung cancer during the follow-up period were more likely to be male and were older than participants without a diagnosis (**Supplementary Table 4**). However, participants with and without lung cancer did not differ in terms of smoking exposure or COPD.

### Association of PRS and PLCOm2012 with lung cancer incidence

**Fig. 1:**
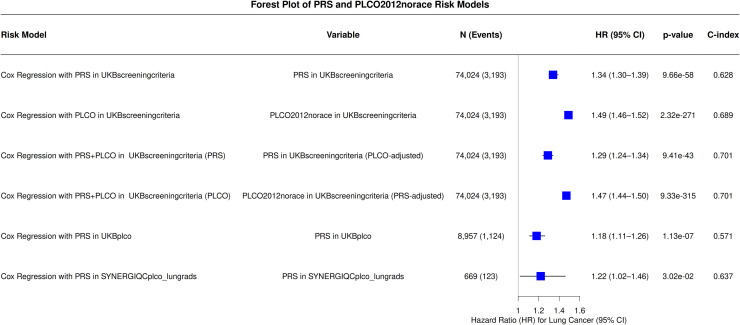
Association Between PRS and Lung Cancer Incidence in Cox Models. Forest plots of hazard ratios (HRs) for PRS and PLCOm2012norace (PLCO) in multivariable Cox proportional hazards models across cohorts. All models were adjusted for age, sex, and 10 first genetic principal components. P-value calculated treating the PRS as a continuous variable. Abbreviations: CI, confidence interval; HR, hazard ratio; PLCO, PLCOm2012norace.

Genome-wide PRS was independently associated with lung cancer incidence in multivariable Cox proportional hazards models adjusted for age, sex, and PCs (Fig. 1). In the UKB_ScreeningCriteria_ cohort, both the PRS (HR per SD=1.34, 95% CI: 1.30–1.39; P = 9.66e^−58^) and PLCOm2012norace model (HR per SD=1.49, 95% CI:1.46–1.52; P = 2.32e^−271^) were significantly associated with lung cancer incidence. These associations remained true after mutual adjustment (PRS_adjustedPLCOm2012norace_ HR per SD=1.29, 95%CI: 1.24–1.34; P = 9.41e^−43^; PLCOm2012norace_adjustedPRS_ HR per SD=1.47, 95% CI: 1.44–1.50; P = 9.33e^−315^), indicating that each provides complementary predictive information. In UKB_PLCO_, each SD increase in the PRS was associated with an 18% increased risk of lung cancer incidence (HR per SD=1.18, 95% CI: 1.11–1.26; P = 1.13e^−7^). In SYNERGIQC_PLCO_LungRADS_, the effect size was similar, but estimated with less precision (HR per SD=1.22, 95% CI: 1.02–1.46; P = 3.02e^−2^).

### Discrimination of predictive performance

**Fig. 2:**
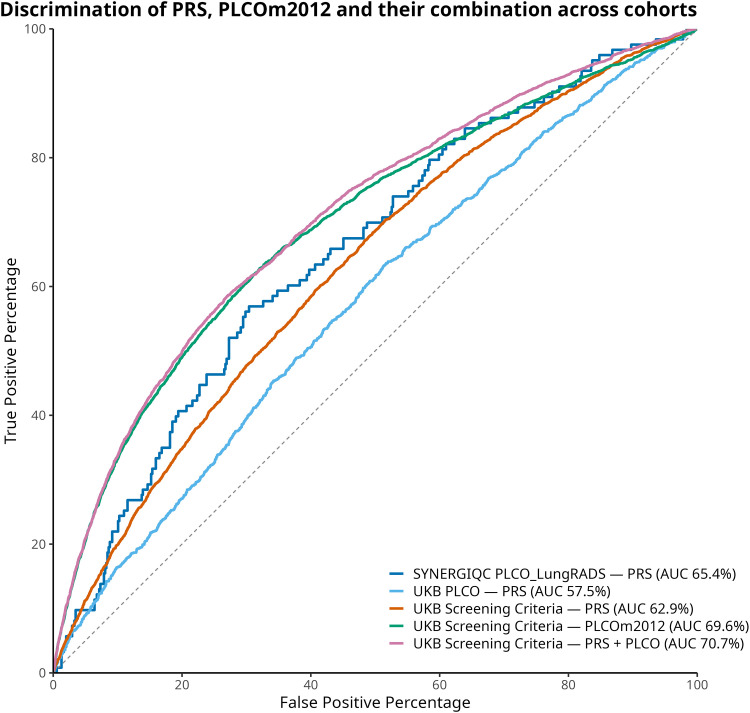
Discrimination of Lung Cancer Risk Models in UK Biobank and SYNERGIQC_PLCO_LungRADS_. Receiver operating characteristic (ROC) curves comparing model (adjusted with age, sex, and first 10 ancestry principal components) performance across cohorts. Shown are AUCs for PRS-only models, PLCOm2012norace-only models, and the combined models. Abbreviations: PLCO, PLCOm2012norace.

**Fig. 3:**
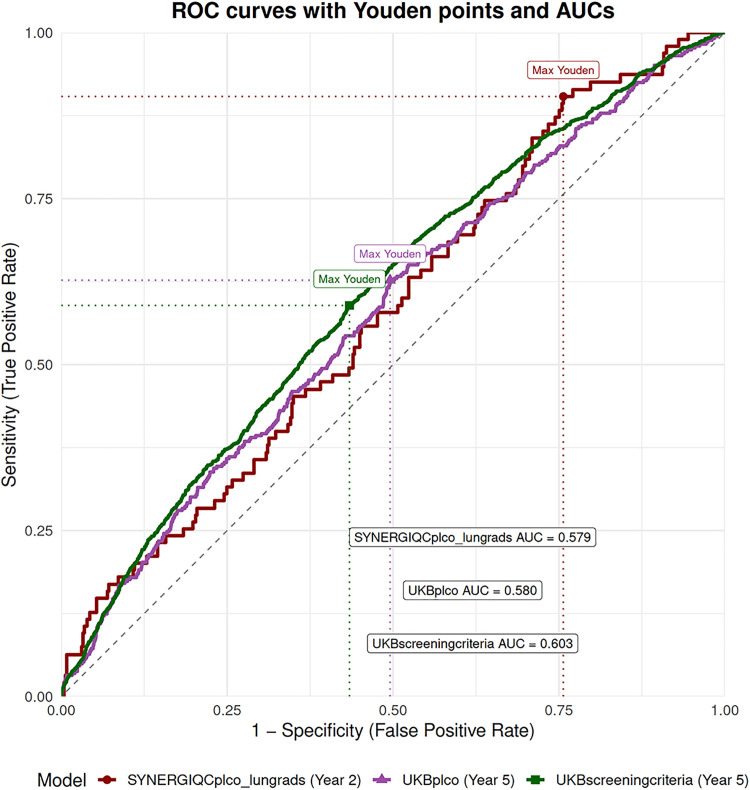
Time-Dependent AUC for PRS Prediction of Lung Cancer. Time-dependent ROC curves for PRS models evaluated at 2 years in SYNERGIQC_PLCO_LungRADS_ and 5 years in UK Biobank.

Receiver operating characteristic (ROC) analyses demonstrated that the PRS modestly improved discrimination both pre- and post-screening (Fig. 2). PRS alone in a model adjusted for age, sex, and PCs yielded AUCs of 0.629 in UKB_ScreeningCriteria_, 0.575 in UKB_PLCO_, and 0.654 in SYNERGIQC_PLCO_LungRADS_. In UKB_ScreeningCriteria_, integration with PLCOm2012norace increased AUC to 0.707 (likelihood ratio test P = 6.85e^−27^ vs. PLCOm2012norace AUC 0.696). Time-dependent ROC analysis (Fig. 3) confirmed stable performance over clinically relevant time horizons. At 5 years in UKB_ScreeningCriteria_, the AUC increased to 0.603 (Youden index=0.155, 84th-85th percentile of the PRS), and in UKB_PLCO_, the AUC remained similar to 0.580 (Youden index=0.132 42nd-43rd percentile of the PRS); at 2 years in SYNERGIQC_PLCO_LungRADS_, the PRS Youden yielded an AUC of 0.579 (Youden index=0.147 45th-46th percentile of the PRS), suggesting an adaptable threshold based on cohort-specific distributions. **Supplementary Table 5** presents the discriminative performance of the risk prediction models over time in UKB subsets and SYNERGIQC_PLCO_LungRADS_.

### Risk stratification: PRS enables gradient discrimination

Higher PRS values were consistently associated with increased lung cancer incidence (**Supplementary Table 6**). In UKB_ScreeningCriteria_, joint stratification by PRS and PLCOm2012norace showed the poorest outcomes in dual high-risk groups and the best outcomes in dual low-risk groups (log-rank P < 1e^−4^; **Supplementary Figure 2**). In UKB_PLCO_, diagnosis-free probability was significantly lower in individuals with high PRS, with a consistent upward trend in incidence across all PRS quintiles (log-rank P < 0.001; **Supplementary Figure 3**). A similar trend was seen in SYNERGIQC_PLCO_LungRADS_ (log-rank P_PRS Youden_=0.016 and P_PRS>90%_=0.028; **Supplementary Figure 4**) and incidence increased with PRS strata (2.1-fold higher in top decile vs. bottom 90%, P = 0.034). These results demonstrate the effectiveness of PRS, whether used alone or in conjunction with existing risk prediction models, to improve lung cancer risk stratification at different stages of screening.

### Classification accuracy and threshold optimization

We compared the performance of different PRS thresholds (Youden, PRS80, PRS90) across cohorts, and in UKB_ScreeningCriteria_ their combination with PLCOm2012norace (Table 1). In UKB_ScreeningCriteria_, PLCOm2012norace alone balanced performance best (sensitivity 36.5%, specificity 87.1%; false positive rate (FPR) 12.9%). Adding PRS modestly increased sensitivity while keeping acceptable specificity: PRS80/90 + PLCOm2012norace reached ∼42% sensitivity with ∼83% specificity and FPR ∼17%. PRS Youden + PLCOm2012norace maximized sensitivity (44.5%) but lowered specificity (80.4%; FPR 19.6%). In UKB_ScreeningCriteria_, the positive predictive value (PPV) remained below 12%, which corresponds to the low prevalence of the disease. In UKB_PLCO_, PRS selected thresholds yielded high specificity (≥83%; FPR (4.8–16.9%), but low sensitivity (<21%), indicating that the PRS may be more useful as an exclusion tool rather than a primary screening test. In SYNERGIQC_PLCO_LungRADS_, PRS90 gave the best discrimination (sensitivity 38.2%; specificity 74.1%; FPR 25.9%). By contrast, the PRS Youden maximized sensitivity (70.6%) at the expense of low specificity (34.9%; FPR 65.1%), which may be advantageous when prioritizing early lung cancer detection in a high-risk post-screening population. These results suggest that the PRS adds context-dependent value in screening programs by maximizing detection in high-risk post-screening populations (SYNERGIQC_PLCO_LungRADS_), excluding low-risk individuals in clinically selected populations (UKB_PLCO_) and modestly improving triage in broader pre-screening populations (UKB_ScreeningCriteria_).

**Table 1:** Performance Metrics of PRS-Based Lung Cancer Risk Models Across Cohorts.

Risk Prediction Model	Sensitivity	Specificity	False Positive Rate	Positive Predictive Value	Negative Predictive Value	Accuracy
UKB_ScreeningCriteria_						
PRS Youden	29.8	80.0	20.0	6.3	96.2	77.9
PRS80	28.8	82.1	17.9	6.7	96.3	79.8
PRS90	25.6	83.4	16.6	6.5	96.2	81.0
PLCO	36.5	87.1	12.9	11.2	96.8	84.9
PRS80 + PLCO	41.9	82.4	17.6	9.6	97.0	80.7
PRS90 + PLCO	42.1	83.2	16.8	10.1	97.0	81.4
PRS Youden + PLCO	44.5	80.4	19.6	9.2	97.0	78.9
UKB_PLCO_						
PRS Youden	20.2	83.1	16.9	15.3	87.4	74.9
PRS quintile	9.4	93.6	6.4	18.0	87.3	82.5
PRS80	8.8	94.5	5.5	19.4	87.3	83.3
PRS90	6.8	95.2	4.8	17.5	87.1	83.6
SYNERGIQC_PLCO_LungRADS_						
PRS Youden	70.6	34.9	65.1	18.2	85.3	41.0
PRS quintile	58.8	42.8	57.2	17.4	83.5	45.5
PRS80	32.4	78.9	21.1	23.9	85.1	71.0
PRS90	38.2	74.1	25.9	23.2	85.4	68.0

Sensitivity, specificity, and related metrics for PRS (e.g., Youden, PRS80, PRS90) alone or combined with PLCOm2012norace. PRS threshold (predefined PRS optimal threshold derived from Youden's index at 5 years in UKB subsets and 2 years in SYNERGIQC_PLCO_LungRADS_). Bootstrap (200) validation used for unbiased estimates.

### Reclassification indices and net benefit

**Fig. 4:**
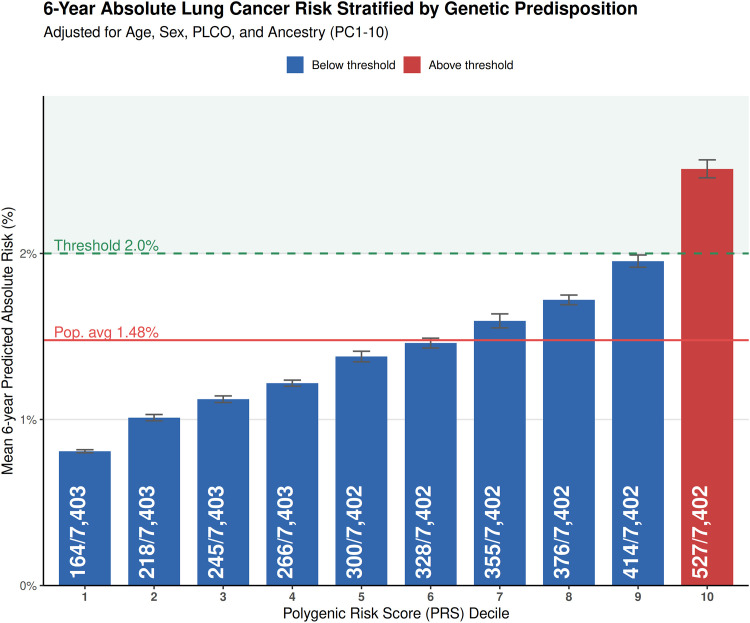
Predicted 6-year Lung Cancer Absolute Risk in UKB_ScreeningCriteria_ Stratified by PRS Decile. Absolute risk was calculated using a multivariable Cox proportional hazards model adjusted for age, sex, 10 ancestry principal components, and the PLCO clinical risk score. The red line represents the mean population risk (1.5%) and the dashed green line the 2% threshold used in PLCOm2012 screening model. Error bars indicate 95% confidence intervals.

Using a fully integrated risk model, we evaluated the six-year absolute risks stratified by PRS deciles in UKB_ScreeningCriteria_ (Fig. 4). Individuals in the 10th decile have a 3.1-fold higher risk (2.51%) than those in the first decile (0.81%). This demonstrates that the model successfully identified lung cancer events that mostly occur in the highest genetic risk tiers. Integrating the PRS into the full clinical model significantly improved the category-based net reclassification index (NRI: 0.032, 95% CI:0.012–0.056), primarily driven by a robust gain in case-detection (NRI+: 0.053, 95% CI: 0.033–0.077). The PRS correctly reclassified 9.2% of incident lung cancer cases (Pr (Up|Case):9.2%) that were otherwise classified as low-risk according to the PLCO criteria. While this increased sensitivity was accompanied by a modest increase in the reclassification of non-cases into the high-risk group (Pr (Up|Ctrl):4.6%), the overall net benefit remains positive, supporting the use of genetic susceptibility to capture high-risk individuals falling outside traditional screening thresholds in the pre-screening population.

**Fig. 5:**
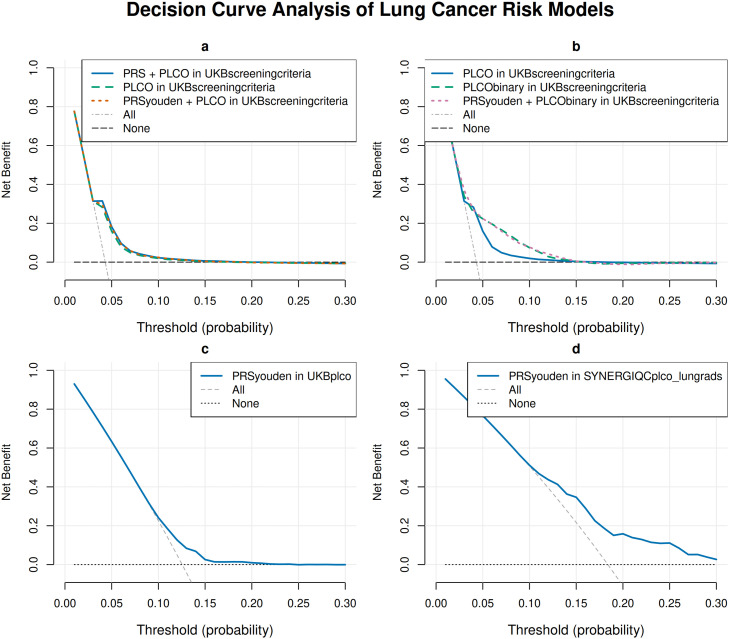
Decision Curve Analysis of Lung Cancer Risk Models. The x-axis is the risk threshold probability that changes from 0 to 1 (right truncated at 0.3) and the y-axis is the calculated net benefit for a given threshold probability. The colorful curves depict the net benefit of the risk model’s selection strategy for screening, whereas the dashed light gray and dotted gray lines display the net benefits in the alternative strategies of “intervention for all” (dashed light gray) versus “intervention for none” (dotted gray) in the data set. (a) presents risk models adjusted for age, sex and PC1–10, including PRS + PLCOm2012norace, PLCOm2012norace only or PRSYouden + PLCOm2012norace in UKB_ScreeningCriteria_. (b) presents risk models including PLCOm2012norace or PLCOm2012 binary (<2% threshold and equal or >2%) only, and PRS Youden + PLCOm2012 binary, age, sex and PC1–10 applied in UKB_ScreeningCriteria_. (c) presents risk models including PRS, age, sex and PC1–10 applied in the subset of participants with PLCOm2012norace risk equal or >2% from UKB_PLCO_. (d) presents risk models including PRS as a continuous variable, age, sex and PC1–10 applied in the SYNERGIQC_PLCO_LungRADS_ cohort. Net benefit was calculated as the standardized difference between true positives and false positives, adjusted for the threshold probability.

Across clinically relevant lung cancer risk thresholds, PLCOm2012norace and PRS / PRS Youden yielded measurable net benefit (Fig. 5). In UKB_ScreeningCriteria_ (Fig. 5a), benefit was negligible, with the model’s curves approaching the "screen none" line beyond the ∼0.15 lung cancer risk threshold probability. When integrating PRS with PLCOm2012norace in UKB_ScreeningCriteria_ (Fig. 5b), the PRS Youden + PLCOm2012norace and binary PLCOm2012norace-alone curves were essentially superimposed across thresholds ∼0.01–0.3, with a minimal incremental net benefit from adding PRS to PLCO in this setting. This suggests that PLCO already captures most actionable risk information in UKB_ScreeningCriteria_. In UKB_PLCO_ (Fig. 5c), benefit was small and limited to a narrow range of thresholds (<∼0.10–0.20). In SYNERGIQC_PLCO_LungRADS_ (Fig. 5d), the PRS Youden curve lay above both hypothetical "invasive procedures for all" and "invasive procedures for none" scenarios between ∼0.1 and ∼0.3 lung cancer risk threshold probability, indicating fewer unnecessary invasive procedures for a similar or higher number of detected cancers within this range. Collectively, these findings suggest that the PRS is most useful as a triage tool for managing lung nodules in high-risk post-screening groups, whereas its incremental value over a strong clinical model for selecting individuals for lung cancer screening appears limited in broader screening-eligible populations. **Supplementary Figure 5** presents DCA of different PRS thresholds (Youden, PRS80, PRS90) in UKB subsets and SYNERGIQC_PLCO_LungRADS_.

## Discussion

Our findings show that a genome-wide PRS adds independent and complementary information to established risk prediction models for lung cancer among ever-smokers eligible for LDCT screening. Across two well-characterized cohorts that differ in screening context and risk spectrum (UK Biobank and SYNERGIQC), the PRS remained a significant lung cancer predictor after adjusting for age, sex, genetic PCs, and PLCOm2012norace scores. Despite the consistent effect sizes observed (HRs ∼1.2–1.3 per SD) and modest but statistically significant gains in discrimination (ΔAUC = +0.011, P < 0.001), this shift represents a non-negligible refinement of individual risk trajectories. In UKB_ScreeningCriteria,_ the model integrating genetic risk factor identified a high-risk stratum in the top PRS decile, with a mean 6-year absolute risk 3.1-fold increase compared to the bottom decile. Notably, this genetic adjustment moved the highest-risk group well above the 2.0% clinical threshold for LDCT eligibility. These results align with recent reports indicating that modern lung cancer PRS can stratify risk within smokers beyond traditional variables and that integrated models may help reclassification of borderline individuals into actionable screening categories [29,30].

Importantly, assessing the net benefit of the PRS using DCA allowed us to pinpoint three observations. First, in UKB_ScreeningCriteria_, adding the Youden PRS to a binary PLCOm2012norace did not materially improve net benefit at screening-relevant thresholds (∼1–4%), indicating that a strong clinical model already captures most actionable risk information. Second, in UKB_PLCO_, PRS alone offered incremental benefit confined to a narrow range of thresholds (<∼10–20%). Third, in SYNERGIQC_PLCO_LungRADS_, a Youden-dichotomized PRS yielded modest but tangible net benefit across ∼10–30% risk thresholds, implying fewer unnecessary invasive additional examinations for similar or greater case detection.

Interpreting DCA in screening is well established and emphasizes net benefit as the decision-relevant quantity across varying preferences and harms [31,32]. The differential PRS performance between the pre- and post-screening cohorts is biologically and design consistent. The absence of DCA improvement when PRS is added to PLCOm2012norace in UKB_ScreeningCriteria_ suggests that the PRS-integrated approach maintains a clinical utility by potentially capturing high-risk individuals who might fall below the threshold of traditional criteria models such as PLCOm2012 or Bach [33,34]. These models rely heavily on smoking duration and intensity and they are not correlated with genetic risk, either biologically or statistically. In UKB_PLCO_ with low event rate and the participant profile filtered based on PLCOm2012, the net benefit of a binary PRS is modestly noticeable at a limited range of thresholds; at such prevalence, PRS primarily acts as an exclusion tool for low-risk participants not identified by the clinical risk model, with minimal additional benefit over "screen none”. In contrast, SYNERGIQC_PLCO_LungRADS_ is enriched with higher baseline risk and LDCT abnormalities (Lung-RADS ≥3) already identified during screening. In this context, additional genetic information may help inform decisions for patients with similar Lung-RADS score but in different genetic risk groups. The integrated model could justify up-classifying the management to an immediate biopsy or PET/CT scan for patients in the top PRS decile. Conversely, clinicians might feel more confident opting for short-term surveillance rather than invasive procedures for patients with a low PRS, especially those with poor performance status and significant comorbidities. For screening programs using risk-model thresholds of 2% 6-year range [23,35], our results suggest that the PRS should be used to manage indeterminate lung nodules rather than as a first-line tool to select participants for lung cancer screening. Guidelines for managing patients with pulmonary nodules support the use of predictive models to classify patients into high- or low-risk groups [36]. However, it has not been clearly demonstrated that current prediction models, such as the Brock model used for initial risk assessment, offer better results than clinical judgment, and are not universally accepted [37]. Future research should focus on developing and validating algorithms that integrate artificial intelligence-based nodule interpretation, the Brock model score, and biomarkers such as PRS. This is clinically relevant, given the ongoing concerns about overdiagnosis and false positives in LDCT programs [38], as well as the need for more effective management of indeterminate pulmonary nodules.

Beyond average effects, PRS gradients in our data identified subgroups with substantially different incidence and timing of diagnosis. Consistent with recent large-scale analyses, individuals in the highest PRS strata experience elevated risk and reach the absolute risk thresholds relevant for screening or lung nodule investigation, whereas those in lower PRS strata accumulate risk more slowly, implications that support personalized screening and management of lung lesions [39]. Taken together, these observations reinforce that germline susceptibility reflects elements of intrinsic risk that are not fully captured by current risk prediction models and provide insight into how to better monitor individuals identified as high-risk before and after screening.

A recent study suggests that risk prediction models for smokers and nonsmokers are essential for lung cancer screening [40]. However, there are controversies regarding the direction of the PRS effect on smoking across ethnic groups [13,16,41]. UK Biobank studies found an increased HR for lung cancer with both smoking status and genetic risk [18]. Zhu et al. [14]. reported that the interaction between smoking and PRS regarding lung cancer incidence was significant. This interaction was not observed in the China Kadoorie Biobank, suggesting smoking-related lung cancer risk differences by ethnicity. Further studies are needed to clarify the interaction between smoking and the PRS, and to determine whether the PRS is more informative in ever or never-smokers.

Strengths of this work include evaluation across two independent cohorts with longitudinal follow-up; a genome-wide PRS derived with state-of-the-art methodology (LDpred2) and robust LD resources; and a multi-metric assessment spanning discrimination, time-to-event performance, and decision analysis. The usefulness of PRS prediction models to enhance screening eligibility and manage pulmonary nodules is shown in three population contexts. Finally, using >70,000 UK Biobank participants bolsters generalizability within European ancestry populations.

Some limitations merit emphasis. First, our analyses were restricted to individuals of European ancestry. The portability of PRS across ancestries remains imperfect due to differences in LD, allele frequencies, and GWAS-detected genetic architecture; multi-ancestry development and recalibration will be essential for equitable deployment [42,43]. Future research must prioritize multiethnic biobanks in order to develop PRSs that will benefit global populations in research and clinical settings, offering an opportunity to promote health equity. Second, the relatively short median follow-up in SYNERGIQC_PLCO_LungRADS_ (∼2.8 years) may underestimate long-term risk separation and may have missed slow growing/low grade lung cancers that warrant mid to long-term clinical follow-up. Third, we focused on PLCOm2012norace; other validated models (e.g., Bach, LLPv2) and their programmatic thresholds are used internationally, and head-to-head comparisons with PRS augmentation warrant further studies [44,45]. Fourth, although our DCA suggests that unnecessary invasive procedures could be reduced while achieving comparable or improved benefits, realized reductions will require validation and prospective evaluation. Finally, we did not incorporate blood-based biomarkers (e.g., ctDNA, small RNAs), which are rapidly evolving and may further enhance early detection and screening programs when combined with imaging and genetic risk factors [46,47].

Collection of a blood sample prior to LDCT, followed by genotyping, would represent an ideal approach for implementing PRS in real-world settings, enabling its use both for refining lung cancer risk prediction in the pre-screening setting and for optimizing the management of indeterminate lung nodules in the post-screening setting. The convergence of risk model-based screening pathways already in practice, inexpensive and scalable genotyping, and maturing PRS methodology support the potential future integration of PRS into screening workflows [48]. Health-economic modeling suggests that risk-stratified strategies that include PRS can be cost-effective under plausible assumptions, though real-world data and randomized designs are needed to establish value, safety, and acceptability [49,50].

## Conclusion

PRS-guided decision making offers context-dependent clinical net benefit depending on the studied populations. In pre-screening populations with strong clinical models, genetic predisposition can reclassify individuals above or below a screening threshold. In high-risk post-screening cohorts, a model with PRS alone can yield modest net benefit across a range of lung cancer risk thresholds. With attention to ancestry equity, benchmarking, and prospective evaluation of PRS-based processes, these results support targeted research on PRS aimed at testing the hypothesis that it could reduce unnecessary diagnostic procedures for indeterminate nodules.

## Funding

This work was supported by the Consortium Québécois sur la Découverte du Médicament (CQDM), SYNERGIQC program in partnerships with AstraZeneca, Pfizer, Biomark and the IUCPQ Foundation through a generous donation from Mr. Normand Lord.

## CRediT authorship contribution statement

**Véronique Boumtje:** Writing – original draft, Visualization, Methodology, Formal analysis, Data curation. **Zhonglin Li:** Methodology, Data curation. **Nathalie Gaudreault:** Writing – review & editing, Resources, Project administration, Methodology. **Victoria Saavedra Armero:** Writing – review & editing, Resources, Methodology. **Dominique K. Boudreau:** Writing – review & editing, Resources, Methodology. **Sophie Plante:** Writing – review & editing, Data curation. **Sabrina Biardel:** Writing – review & editing, Data curation. **Aida Eslami:** Writing – review & editing, Formal analysis. **Simon Martel:** Writing – review & editing, Resources, Investigation. **Sébastien Thériault:** Writing – review & editing, Supervision, Investigation, Conceptualization. **Philippe Joubert:** Writing – review & editing, Investigation, Funding acquisition. **Yohan Bossé:** Writing – review & editing, Supervision, Funding acquisition, Conceptualization.

## Declaration of competing interest

The authors declare that they have no known competing financial interests or personal relationships that could have appeared to influence the work reported in this paper.

## Appendix Supplementary materials

Image, application 1
