# Supplementary material for: Clinical usefulness of polygenic risk scores in risk prediction models for lung cancer screening and lung nodule management
Source: Transl Oncol. 2026 Apr 15;68:102771. doi: 10.1016/j.tranon.2026.102771 (PMC13096907; doi:10.1016/j.tranon.2026.102771)
Supplement: Supplementary file 1 [file mmc1.docx]

**Supplementary Table 1**. UK Biobank data codes for defining lung cancer.

|  | **ICD-9 codes** | **ICD-10 codes** |
| --- | --- | --- |
| Lung cancer | 162, V10.1 | C33, C34, C39.9, Z85.1 |

**Supplementary Table 2**. Comparative Overview of the SYNERGIQC and the UK Biobank Cohort.

| **Variables** | **UK Biobank** | **SYNERGIQC** |
| --- | --- | --- |
| Main objective | Establishment of a large-scale biomedical database for disease prevention and treatment research. | Early detection of lung cancer in high-risk individuals in order to reduce mortality. |
| Enrollment period | 2006-2010. | 2021-2024. |
| Number of participants | 502,166 | 3,542 |
| Eligibility criteria | Age: 37-73 years; Smoking status documented but not a selection criterion; No risk threshold; Participation based on age and consent. | Age: 55-74 years; Current or former smokers; ≥20 years smoking history; <15 years since cessation; PLCOm2012 risk ≥2% over 6 years. |
| Subset of interest | Age: 55 -73 years; Current smokers or former smokers; ≥20 years smoking history; <15 years since cessation. | SYNERGIQC_PLCO_LungRADS_: Low-dose CT with LungRADS ≥3 |
| Total number of participants selected after QC | 74,024; PLCOm2012 risk ≥2% over 6 years= 8,957 | 669 |
| Follow-up period | 2006-2022 | 2021–2025 |
| Lung cancer classification | ICD-9 & ICD-10 codes from health records. | LDCT → LungRADS ≥3 → PET-CT and/or Biopsy → Histopathology. |

This table compares key characteristics of the UK Biobank cohort and the SYNERGIQC. Subsets from the UK Biobank were selected to match SYNERGIQC eligibility criteria, with or without applying the PLCOm2012noRace risk threshold.

**Abbreviations:** *LDCT, low-dose computed tomography; LungRADS, Lung Imaging Reporting and Data System; PET-CT, positron emission tomography-computed tomography; ICD, International Classification of Diseases; PLCO, Prostate, Lung, Colorectal, and Ovarian.*

**Supplementary Table 3.** Number of Genetic Variants and Samples Excluded at each Quality Control Check.

| **Genetic variants** |  |  |
| --- | --- | --- |
| **Quality control parameters** | **SYNERGIQC_PLCO_LungRADS_ GSAv3** |  |
| Illumina GenCall | 1,960 |  |
| Call rate | 6,408 |  |
| Hardy-Weinberg | 599 |  |
| Monomorphic or minor allele frequency | 207,355 |  |
| Remove duplicate variants | 1,753 |  |
| Total unique variants excluded | 218,075 |  |
| Total initial variants number | 730,059 |  |
| Total variants remaining | 511,984 |  |
|  |  |  |
| **Samples** |  |  |
| **Quality control parameters** | **SYNERGIQC_PLCO_LungRADS_ GSAv3** |  |
| Call rate | 0 |  |
| Sex mismatch | 1 |  |
| Duplicate and genetic relatedness | 0 |  |
| Inbreeding coefficients | 1 |  |
| Ethnicity | 3 |  |
| Total unique samples excluded | 5 |  |
| Total initial number of samples | 674 |  |
| Total samples remaining | 669 |  |

**Supplementary Table 4.** Baseline Characteristics of Participants Stratified by Lung Cancer Status Across Cohorts.

| Variable | Level | Participants | Lung Cancer | P-value |
| --- | --- | --- | --- | --- |
| UKBScreeningCriteria (n=74,024) |  | **n=70,831** | **n=3,193** |  |
|  |  |  |  |  |
| Age (mean (SD)) |  | 62.02 (4.03) | 63.05 (3.96) | <0.001 |
| Sex (%) | Female | 34,713 (49.0) | 1,427 (44.7) | <0.001 |
|  | Male | 36,118 (51.0) | 1,766 (55.3) |  |
| Smoking pack-years (mean (SD)) |  | 32.93 (17.43) | 43.22 (22.58) | <0.001 |
| COPD (%) | No | 70,023 (98.9) | 3,081 (96.5) | <0.001 |
|  | Yes | 808 (1.1) | 112 (3.5) |  |
| Family history of lung cancer (%) | No | 60,849 (85.9) | 2,509 (78.6) | <0.001 |
|  | Yes | 9,982 (14.1) | 684 (21.4) |  |
| Time follow-up years* (mean (SD)) |  | 13.76 (0.82) | 7.68 (3.83) | <0.001 |
|  |  |  |  |  |
| UKBPLCO (n=8,957) |  | **n=7,833** | **n=1,124** |  |
|  |  |  |  |  |
| Age (mean (SD)) |  | 65.47 (3.05) | 65.20 (3.12) | 0.005 |
| Sex (%) | Female | 3,099 (39.6) | 414 (36.8) | 0.085 |
|  | Male | 4,734 (60.4) | 710 (63.2) |  |
| Smoking pack-years (mean (SD)) |  | 52.49 (23.36) | 57.11 (24.4) | <0.001 |
| COPD (%) | No | 7,433 (94.9) | 1,046 (93.1) | 0.013 |
|  | Yes | 400 (5.1) | 78 (6.9) |  |
| Family history of lung cancer (%) | No | 4,606 (58.8) | 726 (64.6) | <0.001 |
|  | Yes | 3,227 (41.2) | 398 (35.4) |  |
| Time follow-up years* (mean (SD)) |  | 13.78 (0.83) | 7.32 (3.77) | <0.001 |
|  |  |  |  |  |
| SYNERGIQC_PLCO_LungRADS_ (n=669) |  | **n=546** | **n=123** |  |
| Age (mean (SD)) |  | 64.87 (4.90) | 65.88 (4.57) | 0.037 |
| Sex (%) | Female | 297 (54.4) | 52 (42.3) | 0.020 |
|  | Male | 249 (45.6) | 71 (57.7) |  |
| Education (%) | College graduate | 125 (22.9) | 38 (30.9) | 0.350 |
|  | Some college | 211 (38.6) | 43 (35.0) |  |
|  | Some training after high school | 99 (18.1) | 20 (16.3) |  |
|  | High-school graduate | 47 (8.6) | 13 (10.6) |  |
|  | Less than high school | 64 (11.7) | 9 (7.3) |  |
| Smoking pack-years (mean (SD)) |  | 51.62 (20.59) | 52.24 (22.93) | 0.769 |
| COPD (%) | No | 377 (69.0) | 85 (69.1) | 1.000 |
|  | Yes | 169 (31.0) | 38 (30.9) |  |
| Family history of lung cancer (%) | No | 356 (65.2) | 69 (56.1) | 0.073 |
|  | Yes | 190 (34.8) | 54 (43.9) |  |
| Cancer history (%) | No | 468 (85.7) | 106 (86.2) | 1.000 |
|  | Yes | 78 (14.3) | 17 (13.8) |  |
| Time follow-up years (mean (SD)) |  | 2.90 (0.75) | 1.25 (0.87) | <0.001 |

Baseline demographics and clinical features stratified by lung cancer status in UKBScreeningCriteria (ever-smokers aged 55-73), UKBPLCO (PLCOm2012noRace risk ≥2%), and SYNERGIQC_PLCO_LungRADS_ (high-risk screened cohort with LungRADS 3+). Continuous variables: mean ± SD; categorical: n (%). P-values from t-tests (continuous) or chi-squared tests (categorical).

Time follow-up years * = Follow-up time in the UK Biobank is measured from baseline recruitment to lung cancer diagnosis.

***Abbreviations:*** *COPD, chronic obstructive pulmonary disease; SD, standard deviation.*

**Supplementary Table 5.** Discriminative Performance of the Risk Prediction Models Over Time in UKB subsets and SYNERGIQC_PLCO_LungRADS_.

| Time(years) | AUC | Youden index | Percentile of PRS |
| --- | --- | --- | --- |
| **UKBScreeningCriteria** | | | |
| 5 | 0.603 | 0.155 | 84-85th |
| 10 | 0.597 | 0.140 | 84-85th |
| **UKBPLCO** | | | |
| 5 | 0.580 | 0.132 | 42-43th |
| 10 | 0.566 | 0.101 | 42-43th |
| **SYNERGIQC_PLCO_LungRADS_** | | | |
| 1 | 0.584 | 0.164 | 45-46th |
| 2 | 0.579 | 0.147 | 45-46th |

AUC, Youden index and PRS percentile at the Youden-optimal threshold are reported for SYNERGIQC (1-2 years), UKBPLCO (5-10 years), and UKBScreeningCriteria (5-10 years). Performance was modest but consistent across settings; optimal PRS thresholds varied markedly with cohort composition, ranging from mid-distribution (45-46th percentile) in the enriched SYNERGIQC_PLCO_LungRADS_ cohort to upper-tail values (84-85th percentile) in the population-based UKBScreeningCriteria cohort.

**Supplementary Table 6.** PRS Stratification Performance for Lung Cancer Prediction Across Cohorts and Thresholds.

| Variable | Levels | Participants | Lung Cancer | P-value |
| --- | --- | --- | --- | --- |
| **UKBScreeningCriteria** | **N** | **70,831** | **3,193** |  |
| PRS quintile (%) | 1  2  3  4  5 | 14,422 (20.4)  14,294 (20.2)  14,176 (20.0)  14,074 (19.9)  13,864 (19.6) | 382 (12.0)  511 (16.0)  628 (19.7)  731 (22.9)  941 (29.5) | <0.001 |
| PRS80 (%) | PRS<20%  PRS20-80%  PRS>80% | 14,423 (20.4)  42,544 (60.1)  13,864 (19.6) | 382 (12.0)  1,870 (58.6)  941 (29.5) | <0.001 |
| PRS90 (%) | PRS<10%  PRS10-90%  PRS>90% | 7,239 (10.2)  56,716 (80.1)  6,876 (9.7) | 164 (5.1)  2,502 (78.4)  527 (16.5) | <0.001 |
| PRS Youden (%) | PRS <cut-off PRS ≥ cut-off | **60,703 (85**.**7) TN**  **10,128 (14**.**3) FP** | **2,460 (77**.**0) FN**  **733 (23**.**0) TP** | <0.001 |
| PLCOm2012norace (%) | PLCO <2%  PLCO ≥2% | **62,998 (88**.**9) TN**  **7,833 (11**.**1) FP** | **2,069 (64**.**8) FN**  **1,124 (35**.**2) TP** | <0.001 |
|  |  |  |  |  |
| **UKBPLCO** | **N** | **7,833** | **1,124** |  |
| PRS quintile (%) | 1  2  3  4  5 | 1,286(16.4) 1,404(17.9) 1,582(20.2) 1,682(21.5) 1,879 (24.0) | 126 (11.2)  163 (14.5)  226 (20.1)  270 (24.0)  339 (30.2) | <0.001 |
| PRS80 (%) | PRS<20%  PRS20-80%  PRS>80% | 1,286 (16.4)  4,668 (59.6)  1,879 (24.0) | 126 (11.2)  659 (58.6)  339 (30.2) | <0.001 |
| PRS90 (%) | PRS<10%  PRS10-90%  PRS>90% | 624 (8.0)  6,237 (79.6)  972 (12.4) | 46 (4.1)  887 (78.9)  191 (17.0) | <0.001 |
| PRS Youden (%) | PRS <cut-off PRS ≥ cut-off | **3,493 (44**.**6) TN**  **4,340 (55**.**4) FP** | **396 (35**.**2) FN**  **728 (64**.**8) TP** | <0.001 |
|  |  |  |  |  |
| **SYNERGIQC_PLCO_LungRADS_** | **N** | **546** | **123** |  |
| PRS quintile (%) | 1  2  3  4  5 | 118 (21.6)  106 (19.4)  110 (20.1)  108 (19.8)  104 (19.0) | 16 (13.0)  28 (22.8)  24 (19.5)  26 (21.1)  29 (23.6) | 0.247 |
| PRS80 (%) | PRS<20%  PRS20-80%  PRS>80% | 118 (21.6)  323 (59.2)  105 (19.2) | 16 (13.0)  78 (63.4)  29 (23.6) | 0.084 |
| PRS90 (%) | PRS<10%  PRS10-90%  PRS>90% | 57 (10.4)  442 (81.0)  47 (8.6) | 10 (8.1)  93 (75.6)  20 (16.3) | 0.034 |
| PRS Youden (%) | PRS < cut-off  PRS ≥ cut-off | **135 (24**.**7) TN**  **411 (75**.**3) FP** | **18 (14**.**6) FN**  **105 (85**.**4) TP** | 0.022 |

PRS-stratified lung cancer incidence across UKBScreeningCriteria, UKBPLCO, and SYNERGIQC_PLCO_LungRADS_. Counts (%) for undetermined vs. lung cancer cases by PRS quintiles, PRS80/90, and optimal thresholds. Using time-dependent ROC analysis of follow up, we identified the optimal PRS threshold that maximized the Youden Index (TPR − FPR). Individuals above this threshold (cut-off) were classified as high-risk. P-values from chi-squared tests (all <0.001 except SYNERGIQC_PLCO_LungRADS_ quintiles=0.247). Formulas: Sensitivity = TP/(TP+FN); Specificity = TN/(TN+FP).

***Abbreviations:*** *TN, true negative; FP, false positive; FN, false negative; TP, true positive; TPR, true positive rate; FPR, false positive rate; PRS, polygenic risk score; PLCO, PLCOm2012norace.*


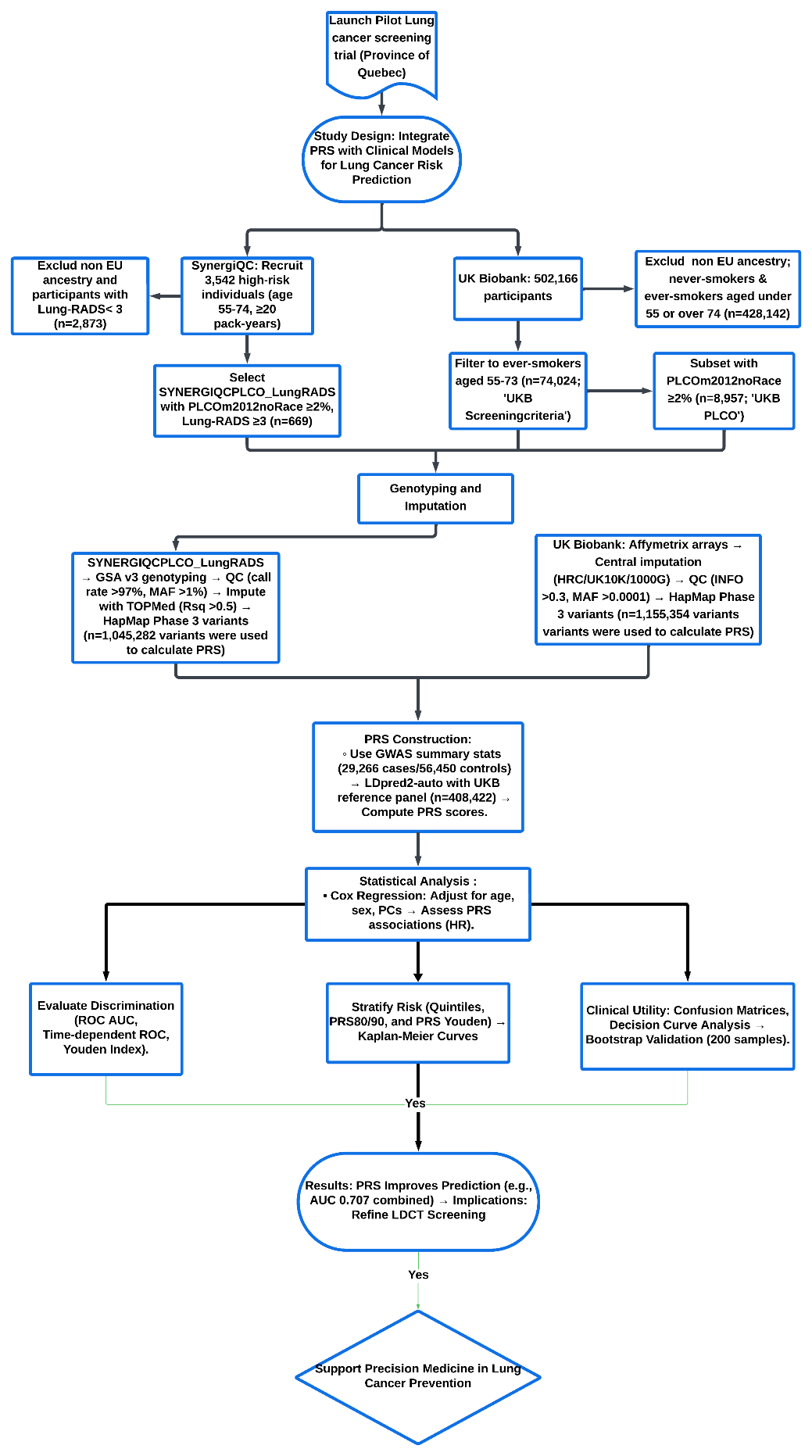


**Supplementary Figure 1.** Workflow for PRS Development and Validation.


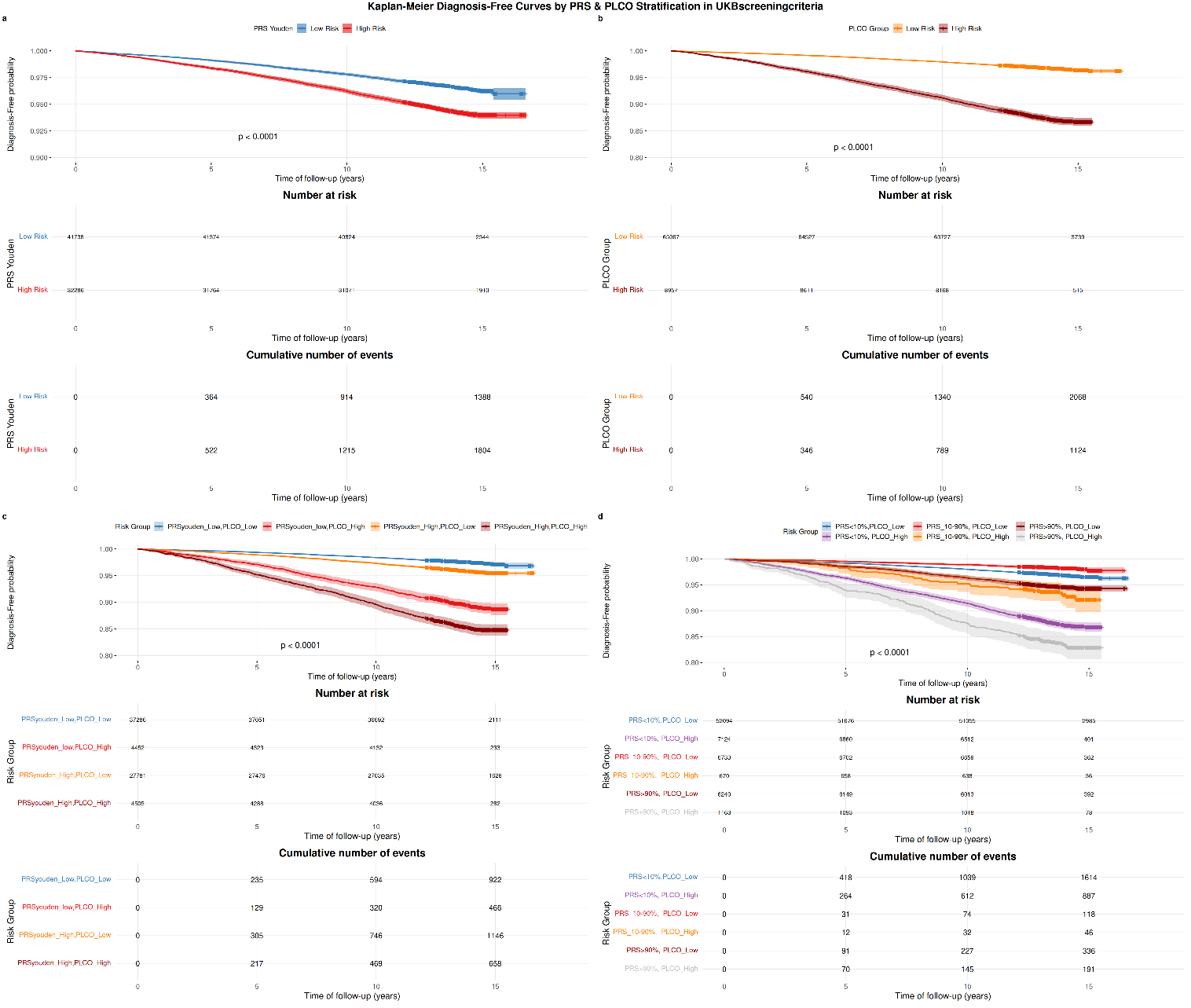


**Supplementary Figure 2.** Joint Risk Stratification by PRS and PLCOm2012norace in the UKBScreeningCriteria Cohort.

Kaplan-Meier diagnosis-free probability by PRS and PLCOm2012norace in the UKBScreeningCriteria cohort. Log-rank tests were used to assess differences between groups. Shaded areas indicate 95% confidence intervals; vertical ticks represent censored observations. All comparisons: log-rank p < 0.0001. (a) PRS Youden group. Participants were stratified according to a predefined PRS optimal threshold derived from Youden's index at 5 years. (b) PLCO group. Participants were stratified according to a predefined PLCOm2012norace groups (Low: PLCOm2012norace≥2%; High: PLCOm2012norace<2%). (c) Risk group. Joint PRS optimal threshold (high vs. low) and PLCOm2012norace (≥2% vs. <2%). The joint categories reveal additive stratification: Low PRS threshold + Low PLCOm2012norace; Low PRS threshold + High PLCOm2012norace; High PRS threshold + Low PLCOm2012norace; High PLCOm2012norace + High PRS threshold. (d) Risk group. PRS90 + binary PLCOm2012norace (PRS <10%, 10–90%, >90% within PLCOm2012norace low/high). Six groups: PRS <10% + Low PLCOm2012norace, PRS 10–90% + Low PLCOm2012norace, PRS >90% + Low PLCOm2012norace, PRS <10% + High PLCOm2012norace, PRS 10-90% + High PLCOm2012norace, PRS >90% + High PLCOm2012norace.

***Abbreviations:*** *PLCO= PLCOm2012norace.*


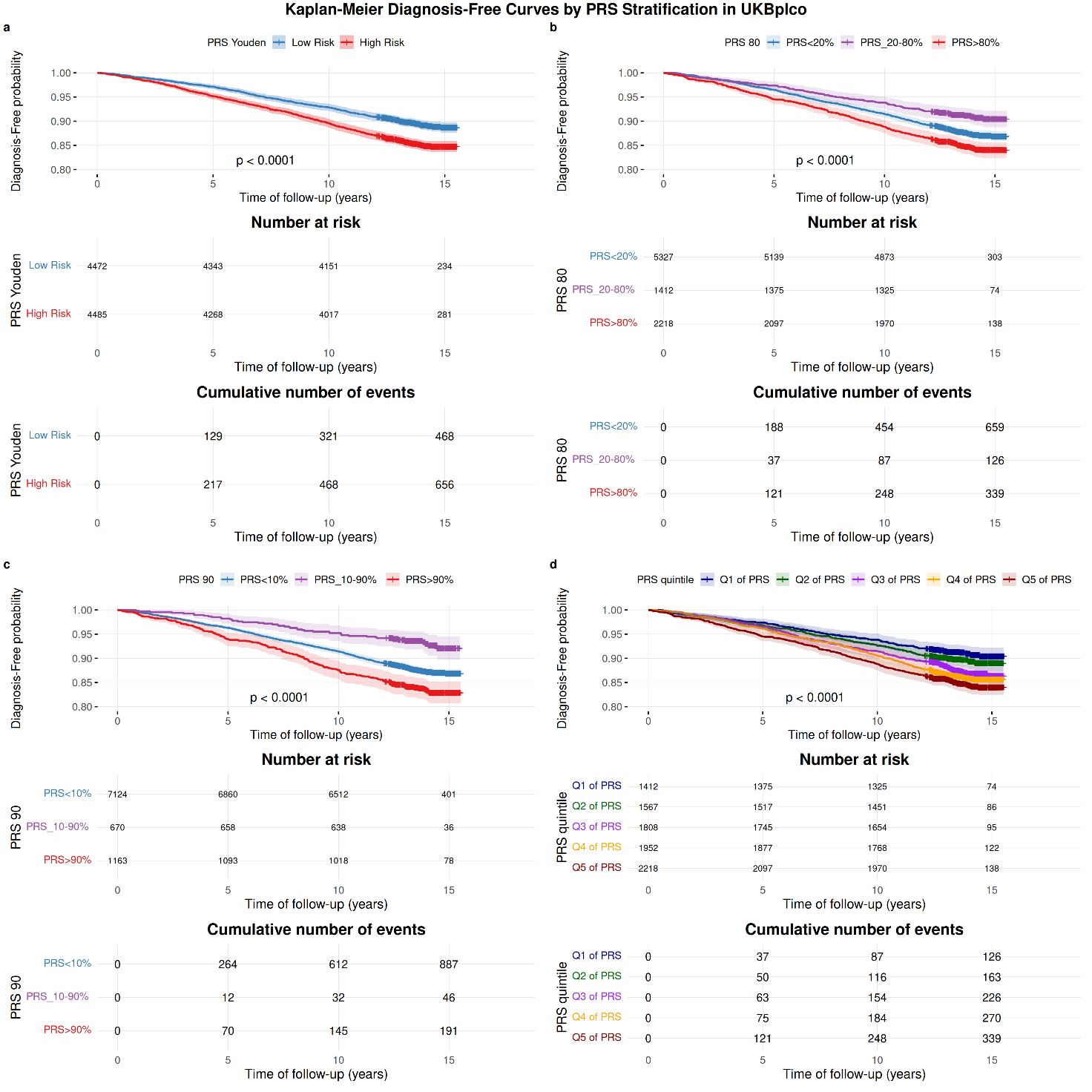


**Supplementary Figure 3.** Lung Cancer Diagnosis-Free Probability by PRS Risk Group in the UKB_PLCO_ Cohort.

Kaplan-Meier diagnosis-free probability by PRS in the UKBPLCO high-risk subset (PLCOm2012norace≥2%). Log-rank tests were used to assess differences between groups. Shaded areas indicate 95% confidence intervals; vertical ticks represent censored observations. (a) PRS Youden Group. Participants were stratified according to a predefined optimal PRS threshold derived from the 5-year Youden index. (b) PRS 80. Participants were stratified into PRS80 categories low: PRS < 20%, intermediate: 20-80%, high: > 80%. (c) PRS90. Participants were stratified into PRS90 categories low: PRS < 10%, intermediate: 10-90%, high: > 90%. (d) PRS quintiles. Stratification of participants by PRS quintiles Q1-Q5.

***Abbreviations:*** *PLCO= PLCOm2012norace.*


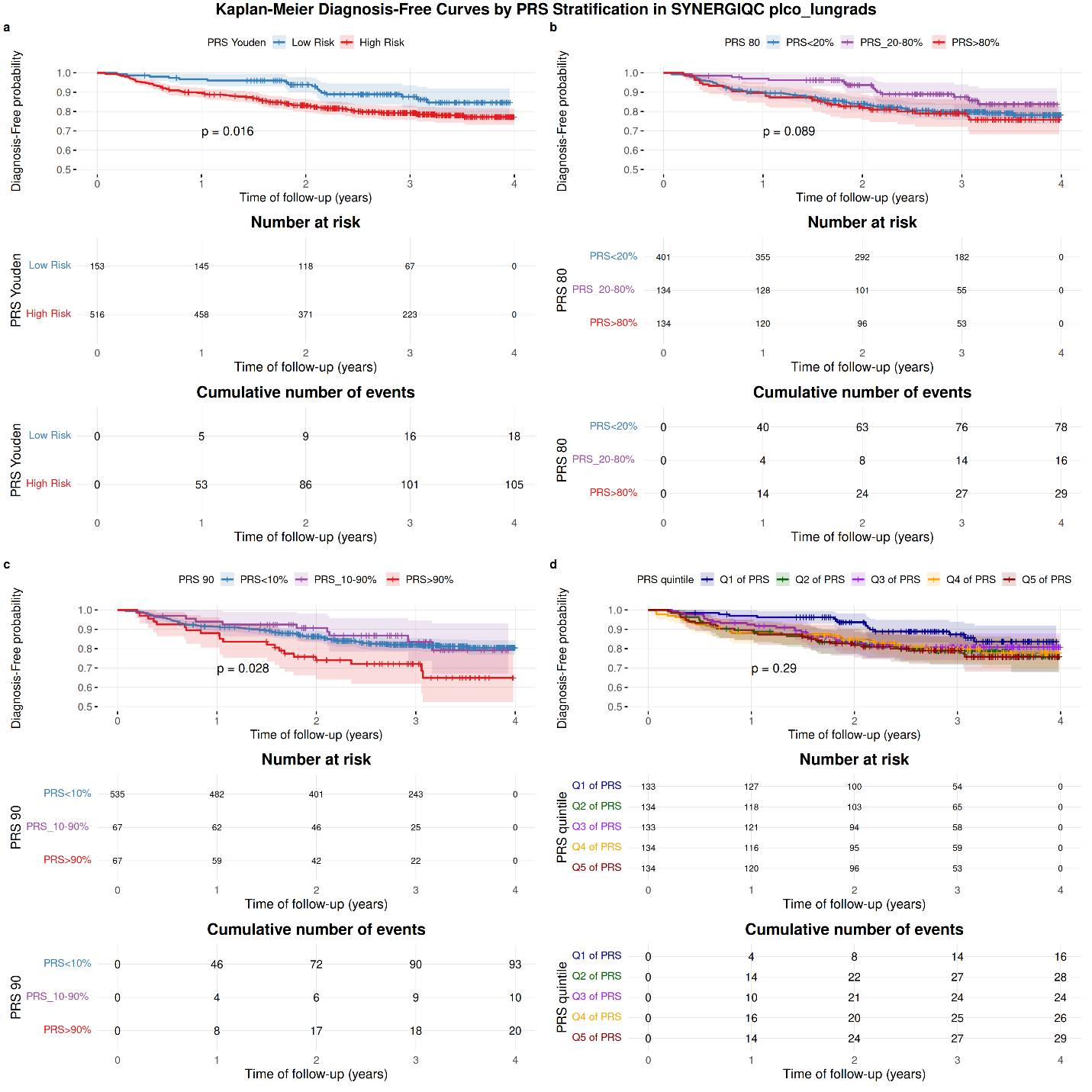


**Supplementary Figure 4.** Kaplan-Meier Diagnosis-Free Probability by PRS Category in the SYNERGIQC_PLCO_LungRADS_ Cohort.

Cumulative diagnosis-free probability in SYNERGIQC_PLCO_LungRADS_ stratified by PRS. Log-rank tests were used to assess differences between groups. Shaded areas indicate 95% confidence intervals; vertical ticks represent censored observations. (a) PRS Youden group. Participants were stratified according to a predefined PRS optimal threshold derived from Youden's index at 5 years. (P= 0.016, log-rank test). (b) PRS80. Participants were stratified into PRS80 categories low: PRS < 20%, intermediate: 20-80%, high: > 80%, (P = 0.089, log-rank test). (c) PRS90. Participants were stratified into PRS90 categories low: PRS < 10%, intermediate: 10-90%, high: > 90%, (P = 0.028, log-rank test). (d) PRS quintiles. Stratification of participants by PRS quintiles Q1-Q5, (P = 0.29 log-rank test).

***Abbreviations:*** *PLCO= PLCOm2012norace.*


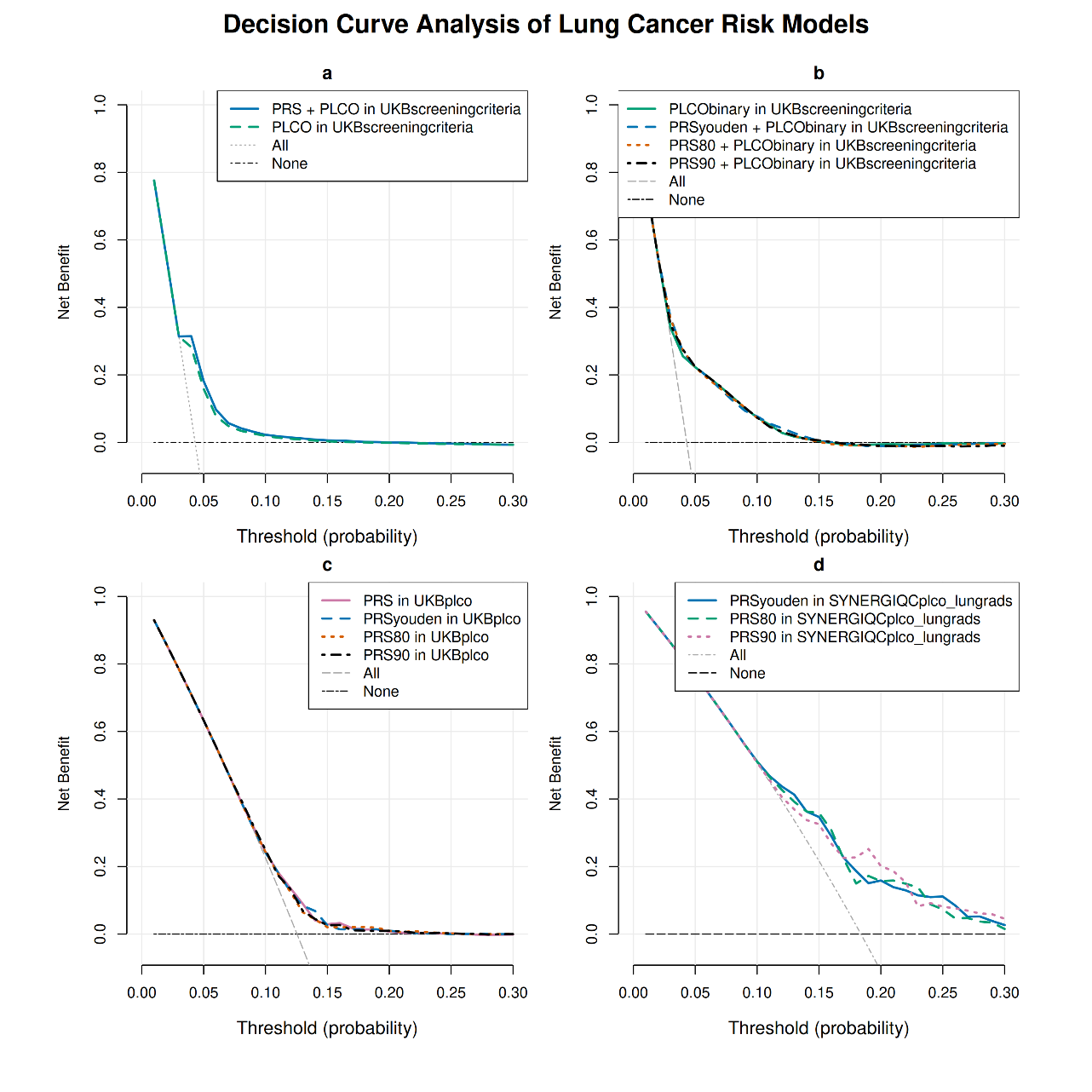


**Supplementary Figure 5.** Decision curve analysis (DCA) of lung-cancer risk models.

The x-axis is the risk threshold probability that changes from 0 to 1 (right truncated at 0.3) and the y-axis is the calculated net benefit for a given threshold probability. (a) present risk models including PLCOm2012norace or PRS Youden + PLCOm2012norace, age, sex and PC1-10 applied in UKBScreeningCriteria (b) the colorful curves depict the net benefit of the risk model’s selection strategy for screening (PRS Youden only, PLCOm2012norace only, and PRS Youden + PLCOm2012norace), whereas the dashed light gray and dotted gray lines display the net benefits in the alternative strategies of “intervention for all” (dashed light gray) versus “intervention for none” (dotted gray) in the data set (c) present risk models including PRS Youden, age, sex and PC1-10 applied in the subset of participants with PLCOm2012norace risk equal or more than 2% from UKBPLCO and (d) present risk models including PRS Youden, age, sex and PC1-10 applied in SYNERGIQC_PLCO_LungRADS_. Net benefit was calculated as the standardized difference between true positives and false positives, adjusted for the threshold probability.

**Appendix A. Adapted from details of the PLCOm2012norace Model.**

The *PLCOm2012noRace* is analogous to the PLCOm2012 model^1^ and differs only in that it has been re-parameterized in the PLCO controls excluding the race/ethnicity variable. To prepare individual 6-year probabilities of lung cancer applying the ***PLCOm2012noRace*** model, the logit (xbPLCO2012norace) has to be calculated first (Step 1) and then it has to be converted into the probability (Probability_PLCO2012norace) through the conversion described in Step 2.

**Step 1 (Equation A.1)**

The logit xbPLCO2012norace =

0.0778895*(Age - 62) +

-.0811569*(Education in 6 Levels - 4) +

-.0251066*(Body Mass Index - 28) +

0.3606082*(Chronic Obstructive Pulmonary Disease) +

0.4683545*(Personal History of Cancer) +

0.584541*(Family History of Lung Cancer) +

0.2675539*(Smoking Status) +

-1.767578*(((Average number of cigarettes smoked per day/10) ^ (-1))- .4021541613) +

0.031949*(Duration of smoking in Years - 39) +

-0.0312719*(Quit Time in years in former smokers - 7) +

-4.536696

**Step 2 (Equation A.2)**

Probability_PLCO2012norace = (exp(xbPLCO2012norace)) / (1 + (exp(xbPLCO2012norace)))

Notes:

Age is in years and is centered at 62.

Education is in 6 levels and is centered at 4. For Education enter the number identifying the highest level obtained: 1 = Less than high school grad; 2 = High school grad; 3 = Post high school training; 4 = Some college; 5 = College grad; 6 = Postgraduate/professional.

Body mass index is weight in kilograms / height in meters squared, and is centered at 28.

Chronic Obstructive Pulmonary Disease (COPD), Personal History of Cancer are coded 1 if present, and 0 if not present.

Smoking status is coded 0 for former smokers and is coded 1 for current smokers. This model was not developed for use in never-smokers.

Average number of cigarettes smoked per day is the measure of smoking intensity and has a nonlinear relationship with lung cancer.

Smoking duration is in years and is centered at 39.

Quit-Time is in years and in current smokers is set to zero.

1. Pasquinelli MM, Tammemägi MC, Kovitz KL, et al. Risk Prediction Model Versus United States Preventive Services Task Force Lung Cancer Screening Eligibility Criteria: Reducing Race Disparities. J Thorac Oncol. 4 August 2020 2020;15(11):1738-1747. doi:10.1016/j.jtho.2020.08.006.
